# Early technology review: towards an expedited pathway

**DOI:** 10.1017/S0266462324000047

**Published:** 2024-01-29

**Authors:** Leslie Levin, Murray Sheldon, Robert S. McDonough, Naomi Aronson, Maroeska Rovers, C. Michael Gibson, Sean Robert Tunis, Richard E. Kuntz

**Affiliations:** 1 EXCITE International, Toronto, ON, Canada; 2 Technology and Innovation, US Food and Drug Administration, Center for Devices and Radiologic Health, Silver Spring, MD, USA; 3 Clinical Policy Research and Development, Aetna/CVS Health, Hartford, CT, USA; 4 Clinical Evaluation and Innovation, Blue Cross Blue Shield Association, Chicago, IL, USA; 5 Department is TechMed Centre, University of Twente, Enschede, Netherlands; 6 Department of Radiology, Radboud University Medical Centre, Nijmegen, Netherlands; 7 Department of Medicine Beth Israel Lahey, Harvard Medical School, MA, USA; 8 Tufts Center for Evaluation of Value and Risk in Health, Tufts Medical Center, Boston, MA, USA; 9 Medtronic Inc., Minneapolis, MN, USA

**Keywords:** technology assessment, biomedical, evidence-based medicine, therapies, investigational, device approval, diffusion of innovation

## Abstract

**Objectives:**

Evidence development for medical devices is often focused on satisfying regulatory requirements with the result that health professional and payer expectations may not be met, despite considerable investment in clinical trials. Early engagement with payers and health professionals could allow companies to understand these expectations and reflect them in clinical study design, increasing chances of positive coverage determination and adoption into clinical practice.

**Methods:**

An example of early engagement through the EXCITE International model using an early technology review (ETR) is described which includes engagement with payers and health professionals to better inform companies to develop data that meet their expectations. ETR is based on an early evidence review, a framework of expectations that guides the process and identified gaps in evidence. The first fourteen ETRs were reviewed for examples of advice to companies that provided additional information from payers and health professionals that was thought likely to impact on downstream outcomes or strategic direction. Given that limitations were imposed by confidentiality, examples were genericized.

**Results:**

Advice through early engagement can inform evidence development that coincides with expectations of payers and health professionals through a structured, objective, evidence-based approach. This could reduce the risk of business-related adverse outcomes such as failure to secure a positive coverage determination and/or acceptance by expert health professionals.

**Conclusions:**

Early engagement with key stakeholders exemplified by the ETR approach offers an alternative to the current approach of focusing on regulatory expectations. This could reduce the time to reimbursement and clinical adoption and benefit patient outcomes and/or health system efficiencies.

## Background

New innovative medical devices are rapidly entering healthcare systems (1). Biomarkers, new diagnostic imaging techniques, robotics, digital health technologies, 3D printing, e-health, and artificial intelligence are, or are on the cusp of, becoming a routine part of health care. For digital health technologies alone, global spending exceeded US$270 billion in 2021 (2) and is projected to increase to US$1,354 trillion by 2030 (3). This is reflected in a rise in healthcare apps to over 400,000 in 2021, with 200 being added each day (3).

The current pathway to adoption in the United States usually begins with developing evidence that satisfies at least one of the Food and Drug Administration (FDA) market pathway authorization criteria, the most common being the 510(k) process which typically involves a detailed comparison of the device’s intended use, indications for use, design, labeling, etc., in addition to performance testing which may require supportive clinical data that demonstrate “substantial equivalence,” i.e. as safe and effective as a currently marketed predicate device or for class II devices without a currently marketed predicate device by granting a de novo request or by establishing a reasonable assurance of safety and effectiveness for premarket approvals (PMAs) for class III devices (1;4).

Regulators are focused on overall “benefit/risk” of the product, i.e., whether the overall potential for health benefit exceeds the overall potential health risks. FDA may grant clearance or approval based on short- to mid-term data with requirements for the manufacturer to conduct post-market studies providing evidence of durable benefit or the absence of significant adverse events over the longer term.

Low-risk medical device technologies that fall within a class I designation and are regulated by FDA are typically exempt from premarket notification requirements but must still comply with general controls, which apply to all classes of medical devices. General controls include but are not limited to provisions that relate to establishment registration and device listing, prohibitions against adulteration and misbranding, records and reports, and good manufacturing practices, unless the device is expressly exempt from those requirements.

The process from regulatory marketing authorization to coverage decision-making and health professional adoption too often lacks a coherent evidence-based approach, because often the data/evidence needed may be different for each stakeholder group. This may lead to evidence development late in the technology development process causing further delays in adoption and patient access(5;6).

In Europe, the pathway to adoption begins with regulatory approval by the European Union (EU) Medical Device Regulation (MDR) which aims to ensure an acceptable standard of safety and quality for medical devices as well as standardizing data and technological advances through a EU database (EUDAMED) (7). The MDR new rules change the type, quantity, and quality of evidence to be generated by the manufacturers, especially of those producing high-risk technologies that are affected by the MDR (8). The recently approved Regulation EU 2021/2282 policy on health technology assessment (HTAR) (7) aims to improve the availability by EU patients to innovative health technologies including medical devices in part by ensuring an efficient use of resources and strengthening the quality of HTA across the EU. The Regulation will apply from January 2025.

There is a need to improve the tools and methods to expedite the adoption of new technologies through meeting the evidentiary needs of regulators, health professionals, payers, and other stakeholders while simultaneously reducing the risk for investors by informing them of the extent to which the technology meets early expectations of these broader stakeholder groups. There is a desire by innovators to engage with healthcare managers early in the development of medical technologies to discuss responsiveness of their innovation to system-level challenges and how they consider the level and intensity of care required by their innovation (9).

The focus on regulatory needs increases the risk of clinical trials being conducted without addressing broader stakeholder expectations, which could affect coverage determinations and adoption into clinical practice. For example, not including sufficient Medicare beneficiaries in the clinical trial may result in Medicare not being able to make a positive national coverage determination (NCD). Coverage determinations and adoption into clinical practice are focused on effectiveness, addressing an unmet need, comparative effectiveness measured against relevant comparators and clinical utility (10), while paying attention to current credible evidence-based guidelines and appropriate use criteria approved by professional associations. Payers are concerned with sustainability and continued relevance of healthcare services they fund through the introduction of technologies that improve patient outcomes and the efficiency of provided services. In the United States, there is inconsistency for the use of economic analysis as a decision determinant in coverage and reimbursement decision-making for medical devices (11).

In the case of FDA and the Center for Medicare and Medicaid Services (CMS), separate statutory requirements for regulatory authorizations and CMS coverage determinations implies two separate and almost independent pathways (12;13). For novel (innovative) start-up medical devices with venture capital (VC) financing, it is possible that VCs may wish to establish a more rapid financial return on investment triggered by FDA market authorization, with no plans for commercialization especially when there are early exits to larger strategic companies.

The FDA, payers, and health professionals all pay close attention to the quality of evidence submitted for their review.

In the United States, CMS is statutorily required to determine whether the technology is reasonable and necessary in their beneficiary population as its criteria for coverage, while also focusing on evidence showing improvement in patient health outcomes, which is common to all payers. Medicare typically requires evidence related to their beneficiary population. Unlike CMS, private insurers are not required to use the CMS criteria of “reasonable and necessary” on which to make a coverage determination (Section 1862 (a) (1) (A) of the Social Security Act). To be eligible for coverage by private insurers, the service must be medically necessary and not experimental, investigational, or unproven.

While regulatory market authorization is needed for patient access for FDA-regulated devices, the engagement of payers and health professionals following market authorization by the FDA is guided by their evidence expectations relating to well-designed and statistically powered clinical trials that address comparative effectiveness based on outcomes, target populations and comparators relevant to their expectations including unmet need. At times, these expectations cannot be met, and coverage determinations are then based on the best available evidence and indirect comparisons. However, whenever possible, these expectations should be made known prior to conducting clinical trials to satisfy regulatory requirements to address these expectations. The ecosystem in which the technology is expected to perform including its place in treatment pathways and whether it should be deployed as a substitute, adjunctive, or additive technology to comparable technologies could affect clinical trial development and economic considerations. It is also important to address the challenge of aligning trial designs which intentionally attempt to minimize confounding through selection bias, to maximize the observed treatment effects vs. real world practice where there may be interest in performance within subgroups that may have multiple comorbidities or be less likely to participate in a trial. Addressing payer, health professional, and patient expectations early in the medical device technology development lifecycle could improve the likelihood of an expedited positive coverage determination and adoption by health professionals. The risk of first-pass rejection by payers is increased if the submission for coverage determination is based on evidence to support regulatory approval and may lead to delayed or failed adoption and increased developer costs. Furthermore, each payer has their own decision-making process, often lacking transparency. This may make it difficult to anticipate what evidence will be sufficient to support reimbursement coverage.

The dissociation between regulatory market authorization and coverage determination can have financial consequences. For example, in 2010, the average cost for a pivotal trial to address 510(k) expectations was $24 million, and for a class III medical device going through PMA, the cost was $94 million (14). Undertaking the regulatory pathway without considering the expectations of health professionals and payers is potentially wasteful and may result in a more lengthy coverage decision-making process and professional guideline development/adoption.

Medical devices with 510(k) clearance are more likely to face coverage restrictions by CMS which often adds conditions such as restricting coverage to patients with the most severe disease (15). Moreover, widespread adoption beyond CMS coverage is hindered by the fact that CMS reimbursement does not guarantee coverage/reimbursement by private insurers (16).

It is assumed that small- and medium-sized device enterprises (SMEs) with limited resources are most likely to prioritize satisfying well-described regulatory expectations without understanding those of payers and health professionals, given the immediacy of getting their product to market. Even for small companies, strategically seeking to be bought out early by a larger strategic company to further develop and market new technologies, evaluation by appropriately designed and statistically validated clinical trials that focus on relevant comparators, outcomes, and target populations are most likely to gain attention.

There are several initiatives aimed at early payer engagement. In 2011, the FDA and CMS set up a pilot parallel review process to decrease the time between FDA’s approval and a NCD by CMS (17). In October 2016, the FDA and CMS published a notice in the Federal Register announcing the indefinite extension of the Parallel Review Program. By 2017, only two biomarker devices for cancer diagnosis had been approved through this program (18). In 2016, the FDA Center for Devices and Radiological Health established a Payor Communication Task Force to invite broader payer input into gathering clinical evidence to support coverage decisions while also helping SMEs in their communications with public and private payers (19). CMS also encourages companies to seek their input prior to pivotal trials for FDA approvals through closer interaction between CMS, FDA, and the National Institutes of Health (20). Other examples of payers sharing their expectations with companies to improve positive coverage and funding determinations include the Blue Cross Blue Shield Association (BCBSA) Evidence Street Program (21) and the National Institute for Health and Care Excellence England Early Scientific Advice Program (22). It is not known how successful these initiatives have been in informing companies of payer expectations for non-drug-related technologies.

We present EXCITE International’s experience as an example of how early engagement with payers and clinical end-users can potentially affect the further development of technologies in ways that are more relevant to coverage determination, diagnostic and treatment guideline development, and patient access. Outcomes arising from these processes are provided.

## The EXCITE approach to early engagement and evidence development

A multi-stakeholder approach to premarket (early) evaluation of medical technologies was developed in Ontario, Canada, in 2012 in which stakeholders likely to influence the pathway from innovation to adoption engaged with companies early in technology development to inform them of their expectations (23). This experience, which followed a successful program that aligned evidence to policy decision-making for adoption of medical devices (24), led to the creation of a nonprofit organization, EXCITE International, in 2016.

While EXCITE set out to provide a premarket collaboration building on existing strengths, programs and health systems in the United States, Norway, the Netherlands, and the United Kingdom (25), most of these efforts to date have been focused on a broad collaboration between payers, health professionals’ regulators, and methodologists in the United States and forms the basis for this publication. The work of EXCITE is driven by four board committees: The Payers’ Advisory Committee (PAC), the Scientific Collaboration, both of which are outlined below, the Advisory Council, and the Industry Advisory Committee. Details of funding for EXCITE are presented in the Funding Statement at the end of the manuscript.

## EXCITE committees

### Payers advisory committee

The PAC influences every aspect of EXCITE’s activities. Its membership comprises senior decision-makers from payers in the United States and includes representatives from BCBSA, CMS, Aetna, Anthem, Providence, and Bright Health (United States), and the National Health Service (United Kingdom). EXCITE continues to encourage other jurisdictions to participate in this initiative.

Members do not represent the interests of their home organization but instead provide advice based on their experience of applying evidence at the policy decision-making interface. No information may be shared that is not available in the public domain. Confidentiality and proprietary information are protected. Members comply with conflict-of-interest policies, including recusal from a technology review in which there is a vested interest. Advice offered has no bearing on subsequent policy decision-making by any payer, this being an independent process governed by specific policies and processes in place for each payer.

Early in the development of EXCITE, PAC produced a consensus document (26) setting out high-level payer expectations for evidence development leading to coverage determinations. This drew on experience from large payers in the United States and from four single-payer universal access health systems and demonstrated similar expectations for high-level decision-making.

### Scientific collaboration

The Scientific Collaboration is comprised of experts in clinical trials methodology, evidence development, and statistical analysis. Selected members of the Collaboration are invited to join Panels, depending on the framework of expectations, described below.

## EXCITE processes

EXCITE’s processes are intended to span the pathway from innovation to adoption and extend beyond a one-off approach typically used by similar initiatives. Companies are expected to communicate directly with regulatory authorities and are invited to share these perspectives, if they wish, as part of the ETR to provide a comprehensive approach to evidence development.

The EXCITE approach uses contextualized evidence that informs technology development according to the expectations of payers and health professionals. The patient perspective is considered important. A meaningful, credible process of engagement is under consideration in part informed by discussions with other agencies such as the FDA and the Medical Device Innovation Consortium (MDIC) who are engaging patients in the technology evaluation process. The patient perspective is also considered under the MDR approach in Europe (7).

While engaging patients in various decision-making roles should always be encouraged, care should be taken regarding evaluation tools to ensure the development of valid scientific evidence from this engagement and the level of patient and public engagement in design and reporting (27;28) using a framework such as that developed by MDIC (29).

## Early technology review: a comprehensive premarket engagement

The intent of the ETR is to inform companies of health professional, payer, and patient inputs regarding early expression of interest, commenting on appropriate target populations and comparators and thresholds, advising on unintended consequences, and helping appreciate changes in patient outcomes and/or health system efficiencies most likely to bring about change in practice and/or funding. The ETR also allows companies and stakeholders to understand more fully whether the technology addresses unmet needs and advises the company regarding further product development and potential facilitators and barriers to adoption.

The ETR provides an opportunity for companies to engage with payers, health professionals, and methodologists (“stakeholders”) usually at the proof-of-concept stage but at any stage up to and including pivotal trial development. The ETR is a robust objective evidence-based platform contextualized by health professionals and payers that sets the stage for a subsequent clinical trial and/or strategic direction for the company.

The ETR process comprises four to six 1.5- to 2-hour virtual meetings over 4 to 6 months, guided by an a priori Framework of Expectations (“Framework”) agreed to by EXCITE, the company, and a panel representing multi-stakeholder interests, which guides the work of the Panel. An early evidence review is undertaken based on the Population, Intervention, Comparator, and Outcomes (PICO) method. This emphasizes the importance of a well-formulated research question to provide clarity about the individual PICO components to establish the agreed-to basis for the evidence review (30). This is followed by an analysis of systematic reviews and meta-analyses undertaken in the last 5 years, complemented by a systematic review of randomized controlled trial (RCT) studies undertaken from the date of the last publication in the analysis. The evidence review forms the starting point for the Panel discussions and is referred to repeatedly during the ETR process.

The Panel is selected from the PAC, the Scientific Collaboration, and leading health professionals relevant to the technology under consideration, all being bound by a non-disclosure agreement. Invited presentations provide additional information as appropriate. A panel chair considered to be a leading authority of the targeted medical condition and/or technology under review is appointed and an expert panel finalized. Panelists are offered an honorarium by EXCITE and are not accountable to the company in any way. Regulatory perspectives are provided by the company at its discretion, reflecting their communication with regulatory authorities. The company participates in all Panel meetings to ensure transparency and to provide information related to the technology under review but is not able to provide any perspective relating to advice developed by the panel. The final ETR is reviewed by the full PAC and Scientific Collaboration and is shared with the company at which point it becomes the company’s intellectual property. While the company may share the content of the ETR at its discretion, it remains confidential to EXCITE and the panel. The ETR does not promote a specific product and reflects an objective, evidence-based approach. The full cost of the ETR is covered by fees received from the company and EXCITE receives no additional revenues for this purpose.

The basic considerations for an ETR Framework are as follows:

- An evidence review that informs Panel discussions.
- Assessment of relevance, based on unmet need and potential impact in improving patient outcomes or health system efficiencies.
- Defining the appropriate target population that optimizes patient outcomes and determine relevance to health professionals and payers, based on the company’s initial perspective and contextualized by health professionals and payers.
- Defining comparator(s) of relevance to health professionals and to payers so that clinical research outcomes allow comparative effectiveness analysis to be determined that are relevant to their expectations. This is essential for professional guideline development and coverage determinations.
- Advice regarding the most relevant outcomes that reflect an improvement in clinical outcomes and/or health systems efficiencies.
- Advice on deployment as a replacement, sequential, or adjunctive technology.
- An assessment of analytical and clinical validity and clinical utility and advice on whether these are sufficient to satisfy health professionals and payers.
- Specified safety issues including weighing benefits and risks and identifying unintended consequences.
- Regulatory requirements shared by the company to establish if these can be included in a broad-based clinical trial that can also address health professional and payer expectations.
- Early health economic modeling to provide estimates of downstream events and costs avoided/incurred and a cost-effectiveness analysis and to determine which uncertainty is the most important to study in a clinical trial. This is optional but recommended if the intent is to enter the United Kingdom, Canadian, or European markets.
- High-level advice on approaches to coverage determination in the United States with specific reference to the technology being reviewed. This includes a review of statutory requirements for CMS coverage, advice on approaches to coverage determination using general and specific codes, and an approach to CMS national versus local coverage determinations (LCDs).
- Positioning within defined clinical pathways contextualized for intended markets.
- High-level advice regarding proof-of-concept and pivotal trial design.

The Framework is expanded according to the technology under consideration.

**Figure 1.**
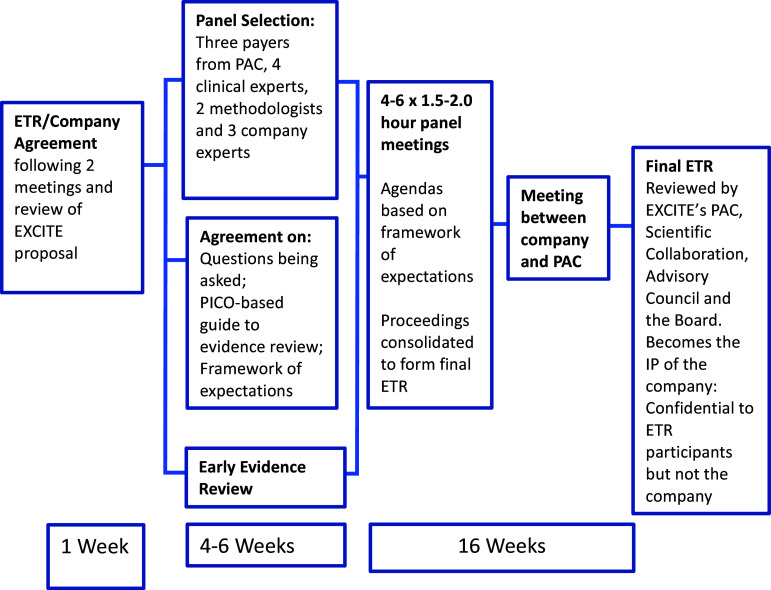
EXCITE evidence technology review process.

The process for an ETR is shown in Figure 1.

To date, EXCITE has, or is in the process of, undertaking eighteen ETRs since the program began in 2017, one of which is not included in this report as it has since been characterized as a drug. This initiative began cautiously and has gradually increased over the past year. Confidentiality precludes a description of technologies reviewed but examples of genericized outcomes from the ETRs are provided below.

ETRs have been conducted on a wide range of medical devices including predictive biomarkers, technologies associated with chronic and surgical wound healing, diagnostic imaging, neuromodulator, sensor-based remote monitoring, point-of-care treatment of acute carbon monoxide toxicity, home-based device to treat a complication from multiple sclerosis, and orthopedic prostheses.

## Genericized examples of advice provided through the ETR process

Thematic genericized examples of outcomes from ETRs considered to potentially affect further technology development are provided below, presented according to their impact on intended outcomes, selection of comparator technologies, advice on clinical trial design, and coverage and reimbursement considerations.

### Examples of Advice based on Intended Outcomes

The most important outcomes of interest to payers and health professionals are routinely provided. Some examples of this advice are provided below.

- Advice was provided against making a claim on a technology outcome that could be difficult to prove.
- Based on an evidence-based review, the effectiveness of an existing gold standard against which the technology was being compared was questioned.
- Advice was provided on acceptable surrogate patient outcomes to shorten a proof-of-concept study. However, surrogate outcomes were not recommended for definitive studies to inform coverage determination.
- A presentation by a statistician was invited regarding the relevance and use of outcomes such as sensitivity and specificity as opposed to negative and positive predictive values to assess the performance of a predictive assay. Payers agreed that sensitivity and specificity would be most likely to inform coverage determination. The company was also advised to include the likelihood ratio as an additional performance metric.
- Advice was provided to use direct patient-related clinical evaluation versus an indirect measure of efficacy.

### Examples of advice based on comparators

- Attention was drawn to the intended use of a comparator as the control arm for a RCT that could have disadvantaged the intervention arm since the latter would have been more likely to alert health workers to the clinical condition in question, biasing emergency room visits which was considered an important outcome.
- Advice was provided to change a comparator that would more closely reflect current practice. This avoided a clinical trial being conducted that would have otherwise reported comparisons irrelevant to health professionals and payers.

### Examples of advice based on clinical trial design

- FDA approval was provided for a technology, based on research using healthy volunteers under controlled conditions. The company was advised to undertake a study on patients exposed to the toxicity in question. This was regarded as a primary requirement for professional use and for coverage determination.
- A company intended undertaking an RCT but the Panel, based on advice from a leading clinical trial methodologist following review, considered that the published research was consistent with a high-quality prospective–retrospective study design and that an RCT would not add new knowledge or improve the existing quality of evidence.
- Following an examination of evidence of improvements in a clinical condition, the Panel advised that the evidence, while demonstrating safety, was hypothesis-generating regarding efficacy since the data were extremely unlikely to meet the expectations of health professionals or payers. The Panel advised that a pivotal clinical trial be undertaken.
- Advice was provided to undertake a sham-controlled study to overcome a placebo effect and considered important to payers and health professionals in reaching a coverage determination and guideline development for the technology whose outcomes were open to subjective bias.
- Advice provided regarding patient selection considering the following options: (i) patients who fail alternative treatment, (ii) patients who are treatment-naïve, or (iii) a combination of (i) and (ii).
- For an RCT aimed at FDA approval, health professionals advised that the clinical trial would create difficulty getting health professional “buy-in” due to a professional practice issue and an adjustment recommended.
- The Panel made recommendations to broaden the scope of trials to include health professional and payer expectations.
- Regarding a technology that could be applied across multiple clinical settings, advice was provided to use risk-associated eligibility criteria across multiple settings rather than adopting a strategy to test the technology through sequential trials for each setting.
- Advice was provided to consider a pragmatic trial design for the comparator arm when an acceptable “gold standard” could not be clearly identified and there were inconsistencies in usual practice across jurisdictions.
- Regarding a technology for which there was an age-restricted comparator, advice was provided to undertake an RCT for patients eligible for use of the comparator and undertake a separate pragmatic trial for the target population in which there was no gold standard of care and in which usual care would constitute the control arm.

### Examples of advice based on coverage and reimbursement considerations

- Information was provided regarding the use by insurers of relevant policies by professional and disease-specific organizations with insights how recommendations by credible bodies may be used to inform the coverage determination process.
- Information was provided on commonalities and differences in approach by CMS and private insurers to coverage determination, expanded on earlier in this article. This was provided to companies whose target populations would require an emphasis by either of these two payment systems.
- Information was provided regarding the importance of health economic modeling to test cost-effective thresholds, especially for drugs and to varying extents for medical devices, in European, United Kingdom, and Canadian health systems and other countries not part of EXCITE’s scope. While economic modeling is not generally expected by most United States payers in the evaluation of medical devices, this decision may be influenced if costs are less than a comparator with similar outcomes in the absence of demonstrated superiority (31).
- Steps were outlined to apply for a code and advice provided on an existing Current Procedural Terminology code under which the technology could be considered.
- The non-specific code 99 was discussed and an explanation of a price threshold for reviewing this code provided together with advice to expect an increasing likelihood of scrutiny and repeated review for approval with increasing cost of the technology.
- Information was provided on hospital diagnosis-related groups, how this is implemented, and funding implications.
- Companies were reminded that payers focus on the quality of evidence, often insisting on RCT evidence (26) or prospective–retrospective studies, the latter especially for predictive molecular diagnostic technologies (32).
- Advice was provided on rental cost arrangements to test the response to a technology prior to payers considering long-term coverage, at which time the device could be purchased or continue to be rented if there has been a demonstrated improvement.
- Payers stressed the importance of understanding compliance with a device if the intent is to purchase it as an insured benefit. This would make it important to evaluate compliance as part of a clinical trial as appropriate.
- Examples of Medicare advice:
  - Information was provided on broad categories Medicare can pay for, further defined by regulations. Companies need to define these into one of the defined benefit categories to assess whether the technology meets the CMS criteria including being reasonable and necessary and improving patient outcomes. Establishing whether a product fits into a benefit category is a threshold question; if there is no category, an alternative to fee for service would need to be sought.
  - Explanation of applied statutory considerations.
  - Advice on seeking one or more LCDs versus a NCD by CMS (33).

## Continuum from ETR to clinical trial

Some companies that have completed ETRs are in the process of engaging through EXCITE and the nonprofit Baim Institute of Clinical Research to develop a clinical trial based on the ETR. Under this arrangement, EXCITE uses its PAC and health professionals who contributed to the ETR to provide any additional input into outcomes, comparator, and target population selection and ensure that the design and quality of the trial addresses their expectations. EXCITE is not involved in the execution of clinical trials under this arrangement. The transition of the ETR to clinical trial development is in the early stages of development. As this process grows, it is intended to also involve other EXCITE-associated clinical trial organizations in the Netherlands, the United Kingdom, and Canada as appropriate. EXCITE has a close relationship with Health Innovation Netherlands which has become an important focus for medical technology development and evaluation, with reach into other countries in Europe. This will allow a more global approach to the work of EXCITE.

## Discussion

The ETR process has the potential to develop an early comprehensive understanding of payer and health professional expectations which could become a platform for undertaking proof-of-concept and pivotal trials that satisfy stakeholder expectations from an early stage in technology development. Examples presented provide insights that could inform further evaluation and development, including mitigating the risk of undertaking clinical trials that might not otherwise have met payer and health professional expectations. This early evidence-based process underpinned by a contextualized early evidence review and comprehensive health professional and payer advice provides an approach to technology development and evaluation that reflects the expectations of stakeholders most likely to affect adoption and diffusion.

The lack of early engagement with payers and health professionals would otherwise have resulted in incomplete preparedness for clinical trial development, a lack of understanding of how the technology addresses unmet needs, and inappropriate or inadequate product development or appreciation of facilitators and barriers to adoption. There is an opportunity, through early multistakeholder engagement, such as described in this article, to add payer and health professional perspectives and expectations to regulation requirements in clinical trial development. This could improve the chances of positive coverage determinations, guideline development, and adoption into clinical practice while responding to expectations of regulatory agencies. It is expected that this approach may improve the quality, relevance, and efficiency of evidence development, decrease costs, reduce market risk, and expedite access to the technology by patients.

We have shown how this process can be transparent to the company and stakeholders while being an objective, evidence-based guide to address expectations of important stakeholders.

Clinical practice guidelines (CPGs) may take time to be adopted into clinical practice, this being affected by, for example, ease of use and strength of evidence. Other determinants associated with the CPG itself may also affect the rate of uptake including specificity, clarity, intended users, and context of practice (34). Nevertheless, a CPG developed by a credible professional organization is an important first step toward adoption and we surmise that understanding the expectations of leading opinion leaders early in evidence development will improve the likelihood of a CPG being subsequently developed.

The feasibility and value-added advice through early engagement with payers and other stakeholders in the development of new medical devices have been described through the ETR process. It will be important to develop criteria for selecting devices that are most likely to benefit from and provide benefit to payers and other stakeholders if this early engagement approach is to be sustainable. As demand grows for ETRs, EXCITE will finalize and apply its selection process through the PAC guideline document (26).

Pivotal trials following the ETR assumes that proof-of-concept studies have been undertaken and a prototype is available for testing. While these steps cannot become part of a pivotal trial, an ETR may provide important insights for a company even during prototype development. An iterative ETR that spans the period from prototype development to proof-of-concept studies and finally a pivotal trial is desirable and should be explored.

As shown in examples provided, premarket identification of factors outside regulatory requirements provides greater transparency to expectations for coverage, payment, and adoption. It may also be more likely to inform guideline development, given the need for quality research that explores relevant outcomes and the involvement of objective key opinion leaders in this process. These developments, combined with regulatory requirements, could mitigate uncertainty for companies and investors.

It is intended that a post hoc review of clinical trial results will be undertaken by engaged stakeholders in the ETR and clinical trial process. This would bring the ETR and clinical trial process to completion with the final assessment being shared by the company with decision-makers involved in coverage determinations and clinical guideline development. Clearly, this cannot interfere with the normal decision-making processes by United States private insurers and professional organizations, but early awareness of these stakeholder expectations is intended to improve the chances of a positive coverage determination and consideration for clinical guideline development. Ideally, this would also reduce the time taken for access for patients and health systems. EXCITE has not yet completed this full cycle, given its recent beginnings.

While it is too early to provide examples of how early engagement can expedite adoption, demonstrated mid-course adjustments to technology development that satisfy health professional and Payer expectations augurs well for this eventuality. The feasibility and advantages of early dialogue between a medical technology developer and major pricing and reimbursement agencies were described by Backhouse et al. (35) who also noted that more experience of early engagement needed to be accumulated, involving a wider range of pricing and reimbursement agencies.

Blankart et al. (36) explored the experience and perceptions of manufacturers to early dialogue using semi-structured interviews and reported that in addition to being feasible and desirable, manufacturers expect early engagement to follow a structured and transparent continuous process and not a one-off discussion. We agree with this perspective, and it is consistent with the approach used for the ETR.

The EXCITE experience has confirmed payers’ willingness to engage upstream in health technology development and evaluation. In our experience, the engagement of health professionals and payers in the ETR process has demonstrated complementarity in terms of evidentiary expectations on target population, outcomes, selection of appropriate comparators, advice on deployment, and how the technology is likely to fit into the ecosystem in which it is designed to function. Until now, many of these factors have not been considered early enough in medical device technology development to inform companies who may face rejection by health professionals and/or payers based on clinical trials designed to satisfy only regulation requirements. Given the costs of undertaking clinical trials, this more comprehensive approach to evidence development may offer cost and time savings to companies who are otherwise unaware of health professional and payer expectations when mounting large expensive clinical trials and potentially shortening the time to patient access, especially to innovative devices for unmet needs.

It is hoped that early engagement will lead to dialogues between payers, health professionals, and innovators, aimed at developing an innovation pipeline with a positive impact on some of the most significant diseases and health system issues.

The use of the ETR as a platform to launch a clinical trial presents an opportunity to complete the participation of payers and health professionals in early evidence development. These stakeholders, who in general are most likely to drive adoption and diffusion, will not necessarily be convinced by findings based on FDA-directed studies designed only to address regulatory expectations. Furthermore, for Class II devices with a predicate technology, regulators may provide clearance based on substantial equivalence to the predicate device without requiring clinical trials. Ideally, to mitigate this downstream risk, premarket clinical trials should address regulatory, health professional, and payer expectations and include credible clinical trial methodological design.

Clinical trials based on results of the ETR are under consideration, and once established, this will cover the full evidentiary pathway from innovation to adoption through a continuous approach, with company and stakeholder input at each step up to but not including final coverage and reimbursement decisions, which are under the independent purview of each payer. EXCITE and other early engagement approaches are intended to reduce risk and streamline the pathway to adoption, much in the same way the helpful, informative, and transparent approach by the FDA improves the chances of subsequent regulatory approval. However, it is too soon to make this determination for early engagement with payers and health professionals.

## Conclusion

Early technology review has demonstrated the feasibility of a novel premarket collaborative approach that informs the early pathway leading from innovation to adoption. Given that the early engagement by stakeholders will most likely impact on the adoption of the technology, this suggested streamlined and risk-mitigating approach is proving to have a positive impact for SME companies, many of whom continue to engage with EXCITE following completion of the ETR. The ETR could become an important platform on which to design and launch clinical trials. It is hoped that this suggested streamlined and de-risked approach toward adoption could benefit patient outcomes and/or health system efficiencies.

Increasing awareness and dialogue through early engagement could foster an innovative pipeline that responds to the needs of health professionals and payers.

## Limitations

Data presented are limited by compliance with confidentiality and the need to protect proprietary information and interests as set out in agreements with companies engaging in the ETR process. The company determines whether to use information based on the ETR. While the process has not been in place long enough to determine the final impact of early engagement on accelerated and de-risked adoption, it is reasonable to assume that early engagement to meet downstream expectations will improve the chances of a positive coverage determination. The experience to date is almost exclusively focused on United States-based payer and healthcare provider expectations.
